# PW01-027 – Predictors and survival of FMF related amyloidosis

**DOI:** 10.1186/1546-0096-11-S1-A80

**Published:** 2013-11-08

**Authors:** E Demirkaya, I Yilmaz, C Acikel, M Saglam, H Unal, M Gok, A Polat, H Cetinkaya, T Eyileten, S Sari, AO Yildirim, Y Oguz, A Vural, JJ Carrero

**Affiliations:** 1 FMF Arthritis Vasculitis and Orphan Disease Research Center, Gulhane Military Medical Academy, Ankara, Turkey; 2 Nephrology, Gulhane Military Medical Academy, Ankara, Turkey; 3 Radiology, Gulhane Military Medical Academy, Ankara, Turkey; 4 Department of Clinical Science, Intervention and Technology, Karolinska Institutet, Solna, Sweden

## Introduction

Amiloidosis is most fatal complication of FMF. Former studies recognize that endothelial functions were severely impaired in patients who have amyloidosis than the patients who have other glomerulopathies. Asymmetric dimethyl arginine (ADMA), the endogenous inhibitor of nitric oxide synthase is possibly a causative or predictive factor in endothelial dysfunction in humans.

## Objectives

Compare the amyloidosis group to only proteinuria group for biochemical, demographic and some other features such as flow-mediated dilation (FMD), to understand which markers may affect or help prediction of amyloidosis. We also evaluated the effects of elevated ADMA levels and impaired FMD responses on the survival time of CVD free period in two distinct groups with severe proteinuria, secondary amyloidosis (SA) versus primary glomerulopathy. We proposed that increased ADMA synthesis in amyloidosis induced endothelial damage may contribute part of the mechanism by which proteinuria increases cardiovascular morbidity and mortality.

## Methods

Study was part of a cohort study. The amiloidosis and proteiuria groups were followed up for predictive factors. All enrolled subjects were evaluated by standard physical examination, chest X-ray, baseline electrocardiogram, two-dimensional echocardiography, and routine biochemical laboratory tests, including liver and kidney function tests and 24-hour urinary protein measurements. FMD and venous blood samples were taken following a 2 week wash-out period, during which time no vasoactive drugs (including colchicines) were given. Measurements of serum ADMA and SDMA were done using HPLC.

## Results

The data of 102 patients with proteinuria due to primary glomerulopathy and 98 patients with amyloidosis due to FMF were assessed. Median age of diagnosis in patients with amyloidosis was 16 (min.-max.: 6-25) and 71.4 % of patients were 18 or younger at the date of diagnoses of amyloidosis. Patients with amyloidosis provided higher levels of SDMA and ADMA (*p* < 0.01) and lower FMD percentage when compared to patients with glomerulonephritis (*p* < 0.01). Inflammatory markers such as high sensitivity C-reactive protein (hsCRP) and pentraxim-3 were statistically different between groups and higher among patients with amyloidosis.

## Conclusion

Inflammatory markers such as hsCRP and pentraxim-3 were statistically different between groups and higher among patients with amyloidosis. The mortality and the cardiovascular event rate was much higher in patients with secondary amyloidosis.

## Disclosure of interest

None declared

